# Depression Symptoms Mediate Mismatch Between Perceived Severity of the COVID-19 Pandemic and Preventive Motives

**DOI:** 10.3389/fpsyg.2021.650042

**Published:** 2021-07-22

**Authors:** Jiwon Park, Seungmin Lee, Sunhae Sul, Dongil Chung

**Affiliations:** ^1^Department of Biomedical Engineering, Ulsan National Institute of Science and Technology (UNIST), Ulsan, South Korea; ^2^Department of Psychology, Pusan National University, Busan, South Korea

**Keywords:** depression, belief, prevention measure, motive, pandemic

## Abstract

The present study monitored changes in beliefs about the coronavirus disease 2019 (COVID-19) pandemic, depressive symptoms, and preventive motives between the first and second waves in South Korea using an online survey administered to 1,144 individuals nationally representative for age, gender, and areas of residence. While participants correctly updated their beliefs about the worsening pandemic situations, the perceived importance of social distancing did not change, and their motives to follow prevention measures shifted toward compulsory rather than voluntary motives. This inconsistency appeared to be mediated by depressive symptoms, such that negative belief changes followed by increased depressive symptoms were associated with the decreased perceived importance of social distancing and decreased voluntary motives. Our data highlights the importance of psychological responses to the dynamically evolving pandemic situations in promoting preventive behaviors.

## Introduction

In December 2019, an outbreak of pneumonia-like acute respiratory syndrome was reported in Wuhan, China, which was found to be caused by a novel coronavirus (SARS-CoV-2) (Zhou et al., 2020a,b). This coronavirus disease 2019 (COVID-19) rapidly spread around the world, and the World Health Organization (WHO) declared the COVID-19 a pandemic on March 11, 2020 (World Health Organization, 2020). At the beginning of the pandemic, there was no available vaccine or identified treatment. Therefore, government officials of many countries emphasized the importance of various non-pharmacological prevention measures, such as social distancing ranging from simple advice to limit contact with others to the total lockdown of the cities and travel restrictions (Chinazzi et al., 2020). Even though vaccines are now available in many countries, it is still considered important to elicit voluntary public cooperation for both vaccination and non-pharmacological prevention measures, including social distancing. It is very unfortunate that even with extensive efforts of government officials on enforcing these prevention measures, most of the countries have been facing non-cooperation of the public (Ryu et al., 2020; Nivette et al., 2021; Wang et al., 2021). Given that the COVID-19 is predicted to be a long-lasting endemic (Hunter, 2020), encouraging individuals to follow the prevention measures still remains a critical challenge across the world.

Besides the effectiveness of social distancing policy, serious concerns have been raised about the negative psychological impacts of the policy, which may induce increased loneliness and other negative effects, including feeling depressed (Brooks et al., 2020; Fiorillo and Gorwood, 2020; Liang et al., 2020; Matias et al., 2020; Torales et al., 2020; da Silva et al., 2021). Enforced social distancing (or prolonged isolation) may influence the affective states and mental health of individuals and alter their motives to follow government policies for preventing the disease. Reduced public cooperation could be a major risk factor for preventing the disease (Kissler et al., 2020; Prem et al., 2020). Thus far, it remains unexamined whether and to what extent the psychological responses of the individual to the constantly evolving COVID-19 situation are related to individuals' intention and motives to follow the prevention measures.

**Figure 1:**
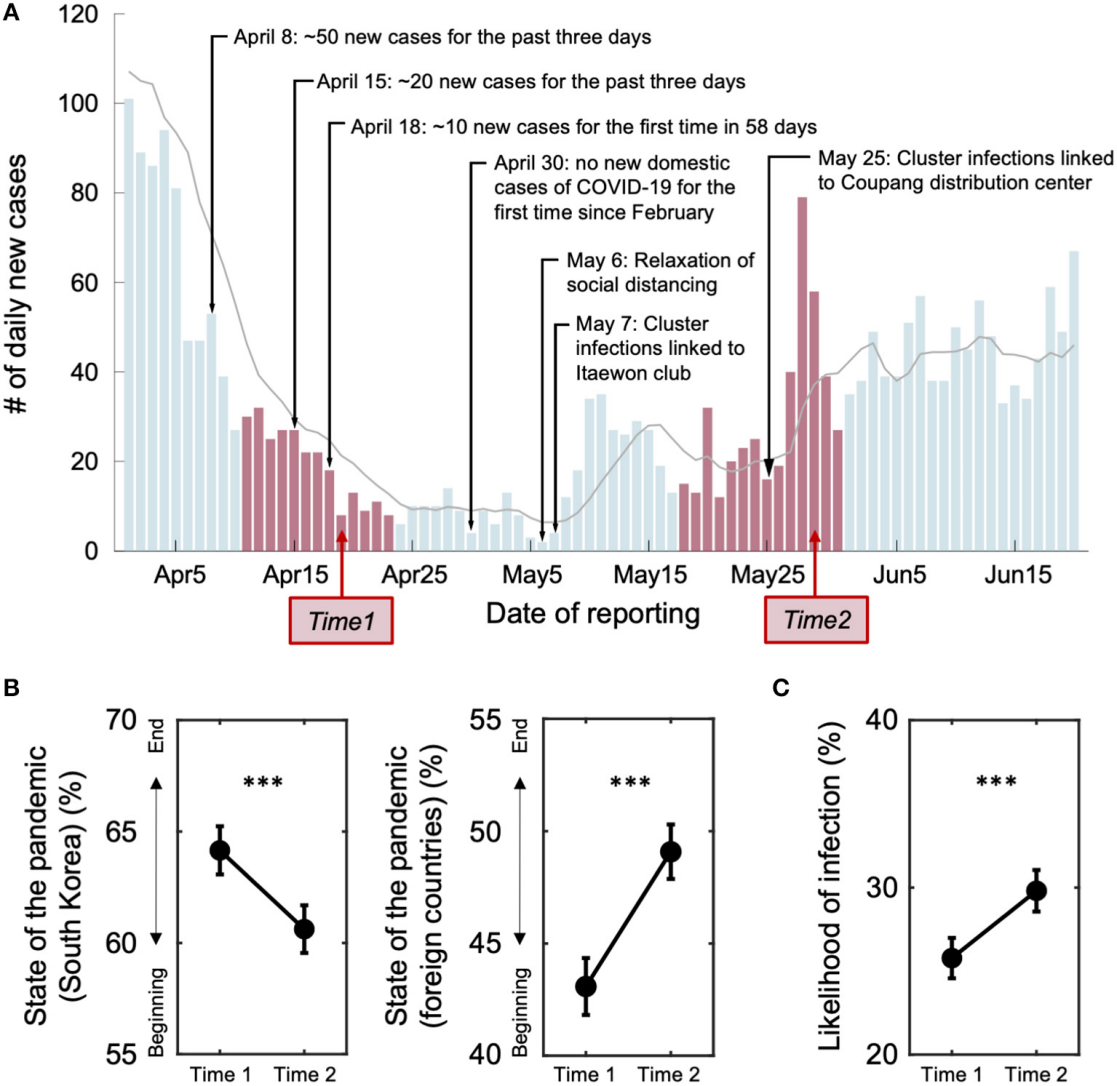
The number of daily new cases of the COVID-19 pandemic in South Korea and the belief changes of individuals between the two time points. **(A)** The number of daily new confirmed cases of the COVID-19 pandemic reflects objective changes in the epidemic status in South Korea. Major news events about the pandemic are labeled. Note that all events relevant to the COVID-19 pandemic before May 6 are positive, whereas those after the date turned negative. Red bars indicate two time periods of data collection: the Time 1 data was collected during the declining phase of the first wave (between April 14 and 20; Time 1 slope = −0.91), and the Time 2 data was collected at the beginning of a second wave (between May 21 and 28; Time 2 slope = 0.73). The numbers of new cases were comparable between the two time points. The gray line indicates seven-day moving averages of the number of new cases. **(B)** At Time 2, people believed that South Korea is further from the end of the pandemic than they expected at Time 1 (temporal distance from the beginning of the COVID-19 pandemic at Time 1 = 64.16 ± 18.58; and at Time 2 = 60.62 ± 18.46). Such a change of belief was specific to South Korea. Participants believed that other countries were getting closer to the end of the pandemic at Time 2 than Time 1 (Time 1 = 43.08 ± 21.89, Time 2 = 49.09 ± 20.83). **(C)** The belief of individuals about likelihood of themselves being infected increased significantly at Time 2, compared with Time 1 [Time 1 = 25.78 ± 20.83, Time 2 = 29.81 ± 21.41; *t*_(1143)_ = −6.42, *P* = 2.02e-10]. Error bars indicate 95% confidence intervals. ^***^P < 0.001.

Here, we examined changes in belief about the pandemic, depressive symptoms, and intention and motives to follow social distancing policy during the drastic changing state of the pandemic between the first (between April 14 and 20, 2020; Time 1) and second (between May 21 and 28, 2020; Time 2) waves in South Korea (Figure 1A; see Supplementary Materials for the COVID-19 pandemic situations in South Korea at the time of research). The clear distinction between the two waves offers an ideal condition to test how individuals react to dynamic changes of the pandemic situation. Given this unique circumstance, we conducted an online survey with a nationally representative sample of South Korean participants for age, sex, and region (*N* = 1,144; Supplementary Figure 1). Data were collected at two time points: one at the decreasing phase of the first wave (Time 1) and another at the increasing phase of the second wave (Time 2). At both time points, we measured the belief of participants about the state of the pandemic (i.e., the temporal distance from the beginning of the pandemic, likelihood of being infected), affective states (i.e., self-reported depressive symptoms), behavioral intention (i.e., the importance of social distancing), preventive behaviors (i.e., frequency of going out, number of people they have met, and average tendency to carry out preventive behaviors), motives (i.e., the reasons of following prevention policies), and other control variables (i.e., demographic information).

Previously, it was shown from experimental studies that the affective responses of the individuals reflect the unexpectedness of the outcomes they experience (Rutledge et al., 2014). Unexpected negative outcomes can be experienced as threatening or uncontrollable, which amplify negative affect and psychological reactance (Brehm and Brehm, 1981; Fogarty, 1997; Crawford et al., 2002; Rosenberg and Siegel, 2018). Based on these previous studies, we hypothesized that negative changes in beliefs about the COVID-19 pandemic situation (believing that the pandemic got worse) would negatively influence the affective states of individuals and decrease their compliance with the prevention measures. Specifically, we predicted that an optimistic expectation from the end of the first wave (i.e., believing that local spreading of the COVID-19 pandemic will end soon) would result in negative prediction error (i.e., change in belief) and subsequent negative affective responses (i.e., increase in depressive symptoms) at the beginning of the second wave, which in turn would reduce voluntary motives and behavioral intention (i.e., the importance of social distancing) to comply with prevention measures recommended by the government.

## Materials and Methods

### Participants

We recruited a sample of 1,500 participants representing the South Korean population in cooperation with a panel-based research agency, Invight (http://www.invight.co.kr). To secure sufficient numbers of participants representing age (20s including 19, 30s, 40s, 50s, and above 60s), sex (male and female), and area of residence (eight provinces including geographically close metropolitan cities), we aimed for a final sample size of 1,000. Therefore, considering ~70% retention rate, we started with a sample size of 1,500 at Time 1. The first data were collected between April 14 and 20, 2020, on which the first wave was on the wane. The second data were collected between May 21 and 28, 2020, at the beginning of the second wave (Figure 1A). A total of 1,144 participants responded to the survey at Time 2 (76% retention rate). Only the participants who completed both surveys (*N* = 1,144; male/female = 583/561, age = 45.04 ± 13.33) were included in the final data analyses (Supplementary Figure 1, Supplementary Tables 1, 2). The research protocol was approved by the Institutional Review Boards of Ulsan National Institute of Science and Technology (UNISTIRB-20-17-C), and all participants electronically provided informed consent.

### Survey Questions Overview

All the questions were in Korean and accessible online *via* computers. At each data collection, participants answered a series of questions about their beliefs, affective states, behavioral intention, preventive behaviors, and motives related to the ongoing COVID-19 pandemic.

#### Beliefs: State of the COVID-19 Pandemic

To measure the perception of individuals about the current state of the COVID-19 pandemic (Figure 1B), we asked the following question (Figure 1B):

- How close do you think South Korea is to the complete end of the COVID-19 pandemic? (0% = *beginning*, 100% = *complete end*)
- How close do you think other foreign countries are to the complete end of the COVID-19 pandemic? (0% = *beginning*, 100% = *complete end*)

We expected that answers to these questions would reflect the perceptions of participants about the severity of the pandemic within the country and outside the country, respectively.

#### Behavioral Intention: the Importance of Social Distancing

**Figure 2:**
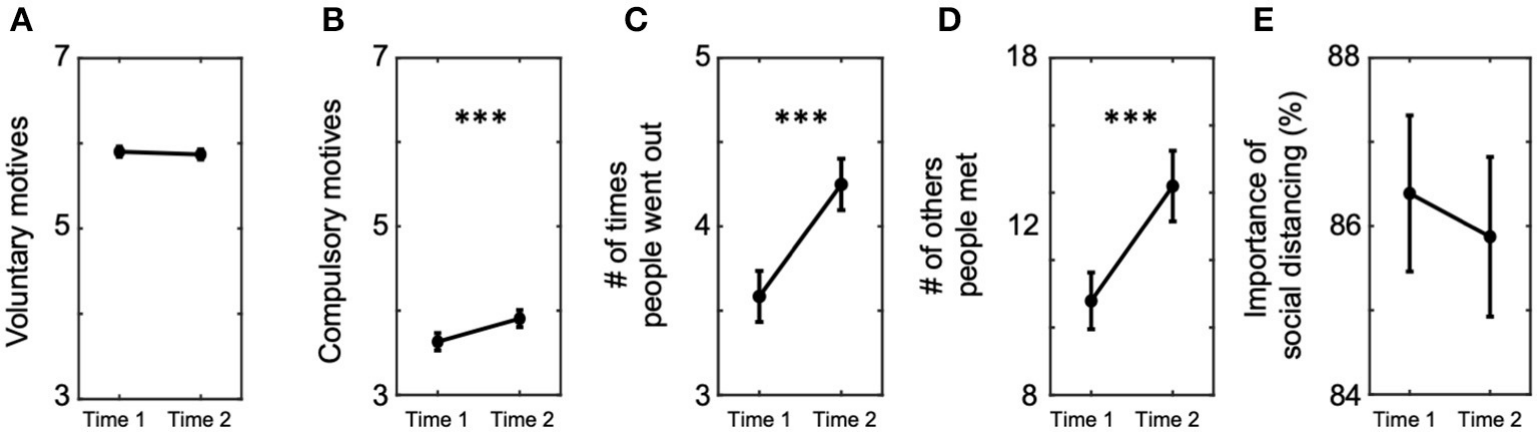
Changes of behavioral intention and motives to follow prevention measures between the two time points. We compared the self-reported behavioral intention and motives of participants. **(A)** Average voluntary motives to follow prevention measures did not change (Time 1 = 5.90 ± 1.02, Time 2 = 5.87 ± 1.01), whereas **(B)** average compulsory motives increased at Time 2 compared with Time 1 (Time 1 = 3.63 ± 1.78, Time 2 = 3.91 ± 1.75). **(C)** Average number of times people went out increased at Time 2 than Time 1 (Time 1 = 3.58 ± 2.59, Time 2 = 4.25 ± 2.61), and so did **(D)** average number of others they met during the past week (Time 1 = 10.79 ± 14.32, Time 2 = 14.19 ± 17.84). **(E)** On the contrary, average perceived importance of social distancing remained the same between the two time points (Time 1 = 86.39 ± 16.02, Time 2 = 85.87 ± 16.34). Error bars indicate 95% confidence intervals. ^***^P < 0.001.

To measure the belief about the importance of social distancing, we asked participants the following question (Figure 2E):

- How important do you think is social distancing? (0% = *not important at all*, 100% = *absolutely important*)

We expected this question to capture the behavioral intention of participants to practice social distancing regardless of the government officials enforcing the policy.

#### Preventive Behaviors: Average Tendency to Carry Out Preventive Behaviors

Participants were asked to self-report their average tendency to follow preventive behaviors (e.g., washing hands and wearing face masks) during two months before Time 1 and Time 2. Participants reported how frequently they followed each preventive behavior listed below in a seven-point Likert scale (1 = *never*, 7 = *very frequently*):

For the past 2 months, even if I did not have any symptoms of sickness,

- I washed my hands or used hand sanitizer whenever I went to work or came back home.
- I covered my mouth and nose with sleeves whenever I coughed or sneezed.
- I did not touch my eyes, nose, or mouth before washing my hands.
- I wore a face mask whenever I visited a medical institution (e.g., hospital, drug stores).
- I wore a face mask whenever I went out.
- I refrained myself from visiting crowded places.
- I avoided meeting people who had symptoms such as high fever or respiratory illness.
- I refrained myself from going out or visiting other cities.

Note that the list above is the preventive behaviors recommended by the South Korean government and, therefore, should be familiar to most of our participants. We also provided an option of “Not applicable” for the cases where participants did not face a certain situation [e.g., people who never visited a medical intuition could choose “Not applicable” instead of selecting “never (1)”]. For the mediation analyses (described below), we formed a composite score by averaging answers to all eight questions, except those that were not applicable. Three individuals who responded “Not applicable” to all eight questions were excluded from the mediation analyses, where the preventive behavior of individuals was included as a predictor or a moderator.

#### Motives: Voluntary and Compulsory Motives Underlying Compliance With Prevention Measures

To examine participants' motives for compliance with the prevention measures recommended by the government (e.g., keeping distance from others and wearing face masks), we asked the following nine questions (Figures 2A,B):

I followed the prevention measures against coronavirus recommended by the government because

- I know that anyone can get infected based on the public information about infectees.
- I am concerned that I may get infected.
- I am concerned that my family members may get infected.
- I am concerned that my friends and acquaintances may get infected.
- I am concerned of broader viral spreading in South Korea.
- I am concerned that my action may negatively affect the groups which I am part of (e.g., workplace, school, or religious group).
- I am concerned of the pandemic becoming more serious than the current status.
- I am afraid of being subject to legal penalties.
- I am afraid that other people may blame my actions when all information is shared by contact tracing.

The first seven items are relevant to viral infection and voluntary motives, and the last two are associated with being forced by law or social sanction. Participants responded on a seven-point Likert scale to indicate the extent to which each question correctly describes why they followed prevention measures (1 = *definitely not*; 7 = *definitely*). For the mediation analyses (described below), we created two composite scores; an average of the first seven ratings is defined as “voluntary motive,” and an average of the last two ratings is defined as “compulsory motive.”

#### Depression Symptoms

We asked participants to report the degree to which they were experiencing depressive symptoms at each time point, using the Korean version of the Zung Self-Rating Depression Scale (SDS) questionnaire (Lee, 1995). The validated Korean translation (Zung, 1965) consists of 20 items where participants are asked to rate how each item applies to them at the time of testing in a four-point scale: a little of the time, some of the time, a good part of the time, and most of the time. Values of 1, 2, 3, and 4 are assigned to these responses, respectively, when the question is worded negatively. The questions that are worded positively were inversely coded. Sum of the assigned values to all 20 questions (raw SDS score) measures depressive symptoms, with its scores ranging from a minimum score of 20 to a maximum possible score of 80. We used the raw SDS scores to measure the self-reported severity of depressive symptoms.

#### Other Measures

In addition, we included the likelihood of viral infection (Supplementary Figure 2), direct measures of violating behaviors against social distancing, and basic demographic information (age, sex, and area of residence). See Supplementary text for details about the questions we used. See Supplementary Figures 9, 10 for correlations among the major variables-of-interest.

### Mediation Analyses

**Figure 3:**
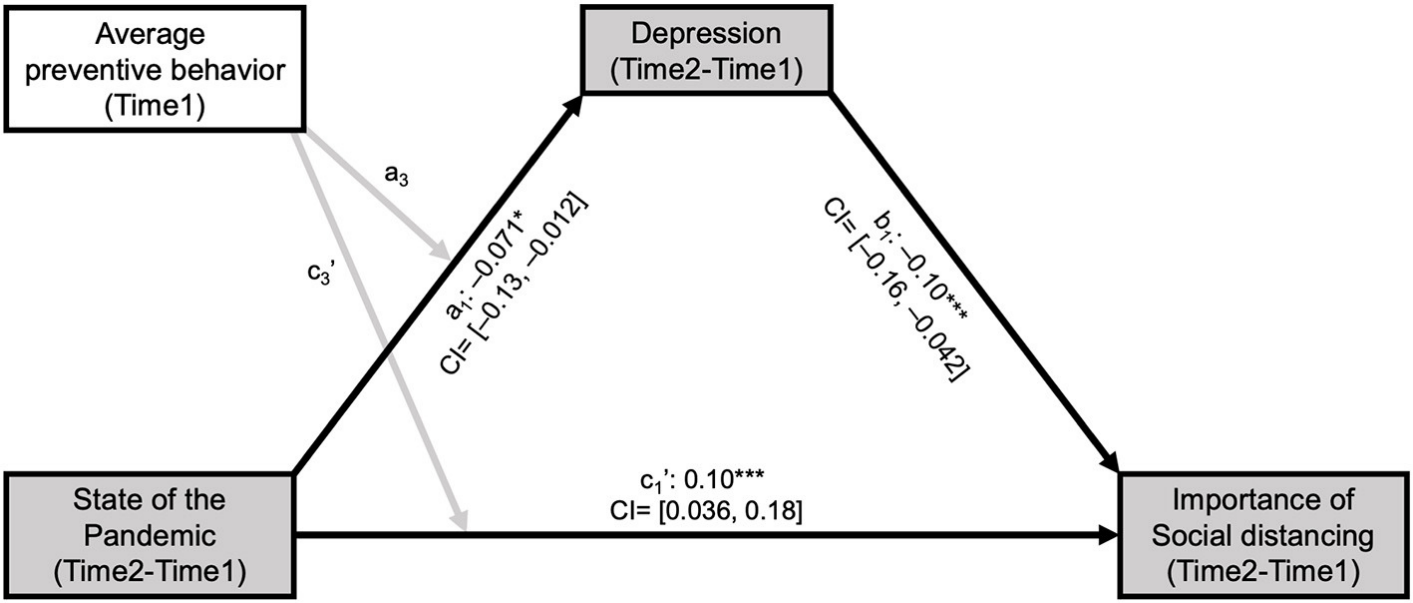
Changes in depressive symptoms mediated the inconsistency between belief about the COVID-19 pandemic state and the perceived importance of social distancing. To examine the moderated mediation effect of depressive symptoms, we set belief about the COVID-19 pandemic state (negative score for Time 2—Time 1 indicates “pandemic got worse”) as a predictor, the average tendency of individuals to follow preventive behaviors (e.g., wearing masks) as a moderator and perceived importance of social distancing as an outcome variable. Change in depressive symptoms between Times 1 and 2 was significantly associated with a change in belief about the COVID-19 pandemic state negatively (a_1_: *t* = −2.40, *P* = 0.016) and with an average tendency to follow preventive behavior before Time 1 positively (a_2_: *t* = 2.39, *P* = 0.017; path not depicted). An increase in the severity of depressive symptoms was associated with a decrease in the perceived importance of social distancing (b_1_: *t* = −3.39, *P* = 0.00072). After adjusting for the mediation effect of change in depressive symptoms, the direct effects of belief change (c_1_': *t* = 3.56, *P* = 0.00038) and average tendency to follow preventive behavior (c_2_': *t* = −1.99, *P* = 0.047; path not depicted) on the perceived importance of social distancing was still significant. Moderated mediation effects of the two predictors (i.e., the interaction between the state of the pandemic and average preventive behavior) on change in depressive symptoms (a_3_: *t* = −0.87, *P* = 0.93) and change in the perceived importance of social distancing (c_3_': *t* = −1.73, *P* = 0.083) were not significant. Black and gray arrows indicate significant and non-significant associations between the components, respectively. ^*^P < 0.05, ^***^P < 0.001; CI: 95% bootstrap confidence interval for each of the standardized beta estimates.

To test whether the effect of belief about the pandemic on behavioral intention is mediated by the affective states of individuals, we analyzed the mediation models using the PROCESS for SPSS macro (model 8 and model 4 therein) (Hayes, 2017). For each subject, four components were entered into the model (model 8; see Figure 3): an initial predictor, a mediator, an outcome, and a moderator that may moderate the relationship between predictor and mediator, and the relationship between predictor and outcome. Perceived change in the COVID-19 pandemic state of South Korea between Time 1 and Time 2 (updates in “Beliefs”) was set as a predictor, change in self-reported severity of depressive symptoms (i.e., affective states) was set as a mediator, and change in the perceived importance of social distancing (“Behavioral intention”) was set as an outcome. We hypothesized negative impacts on the outcome variable to be larger for individuals who experienced larger changes in their beliefs. Moreover, we expected that participants who followed prevention measures more diligently during the first phase of the pandemic would be disappointed more (because they had reasons to expect positive consequences) and thus would show more exaggerated negative impacts (e.g., reducing behavioral intention). Based on this additional hypothesis, the individual tendency for preventive behavior at Time 1 (“Preventive behaviors”) was used as a moderator. In addition, age and sex were entered as covariates to control for potential confounding effects. The significance of the direct and indirect effects was estimated using the bootstrapping method (5,000 bootstrapping samples, alpha level = 0.05). All continuous measures were Z-scored before being entered into the model. Furthermore, we used “model 4” of the PROCESS macro, which examines mediation effects without a moderator, to examine the robustness of each mediation effect (i.e., state → depression → importance, and preventive behavior → depression → importance; see Supplementary Figure 3).

**Figure 4:**
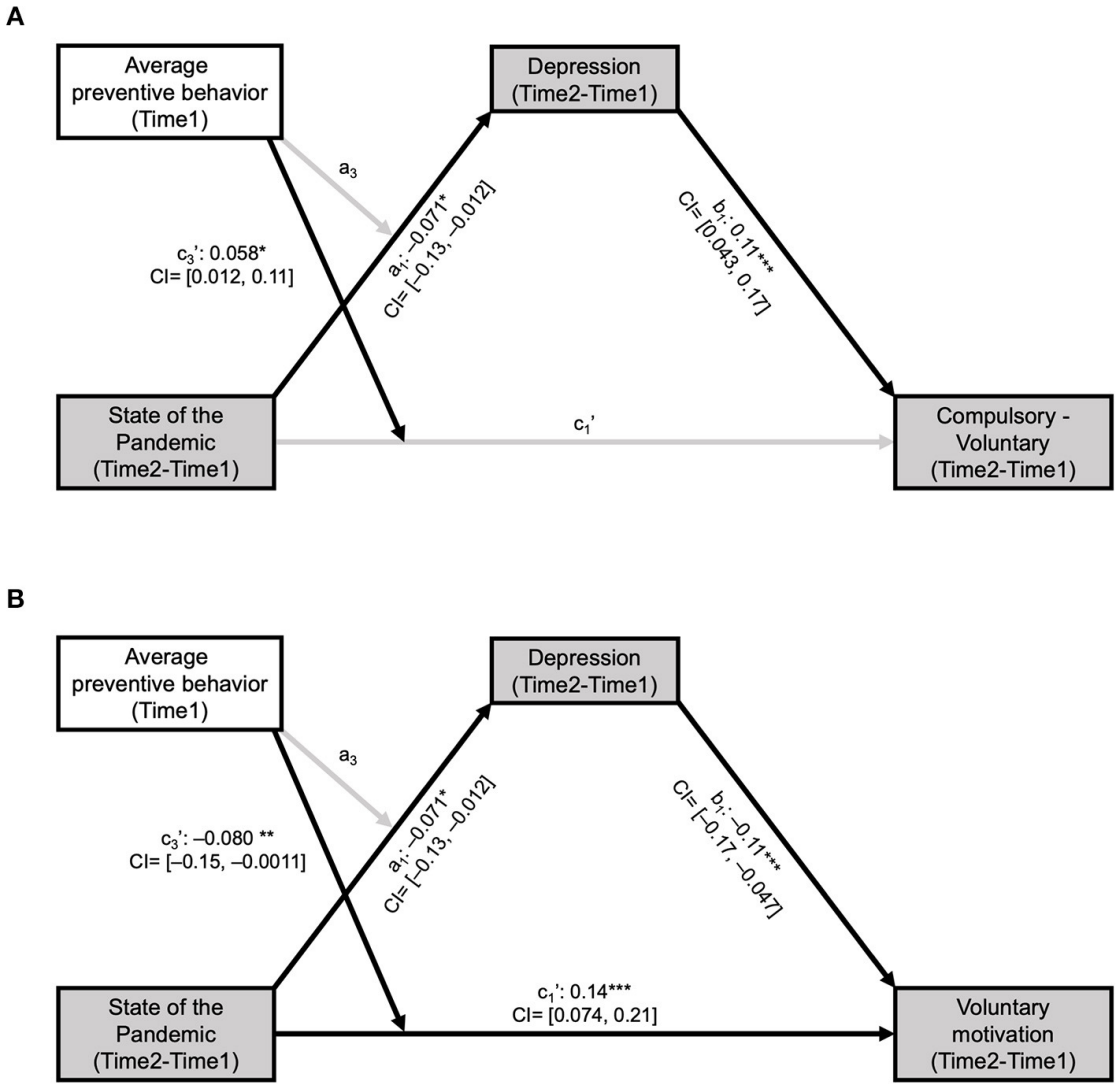
Changes in depressive symptoms mediated the inconsistency between belief about the COVID-19 pandemic state and voluntary motives to follow prevention measures. **(A)** To examine the moderated mediation effect of change in depressive symptoms, we set to change in belief about the COVID-19 pandemic state (negative score for Time 2—Time 1 indicates “pandemic got worse”) as a predictor, the average tendency of individuals to follow preventive behaviors (e.g., wearing masks) as a moderator, and compulsory vs. voluntary motives to follow prevention measures as an outcome variable. Change in depressive symptoms between Time 1 and Time 2 was significantly associated with both change in belief about the COVID-19 pandemic state (a_1_: *t* = −2.40, *P* = 0.016) and average tendency to follow preventive behavior before Time 1 (a_2_: *t* = 2.38, *P* = 0.017; path not depicted). Individuals with increased depressive symptoms showed greater increase in compulsory than voluntary motives [compulsory(Time 2)—voluntary(Time 2)]—[compulsory(Time 1)—voluntary(Time 1)] (b_1_: *t* = 3.66, *P* = 0.00026). After adjusting for the mediation effect of the depressive symptoms of individuals, the direct effects from the belief change and preventive behavior to the motivational change were not significant (c_1_': *t* = −0.25, *P* = 0.80, c_2_': *t* = 0.31, *P* = 0.76; c_2_' path not depicted). Nevertheless, the interaction between the belief change and the average tendency to follow preventive behavior on the motivational change was significant (c_3_': *t* = 2.32, *P* = 0.021). **(B)** Particularly, individuals with increased depressive symptoms showed a greater decrease in voluntary motives (b_1_: *t* = −3.72, *P* = 0.00021). After adjusting for the mediation effect of the depressive symptoms of individuals, the direct effects of the belief change (c_1_': *t* = 4.88, *P* < 0.000010) and average tendency to follow preventive behavior (c_2_': *t* = −3.48, *P* = 0.00051; path not depicted) were both significant. The interaction effect between the two predictors on the changes of voluntary motives was significant (c_3_': *t* = −3.29, *P* = 0.0011) but was not significant on the change in depressive symptoms (a_3_: *t* = −0.087, *P* = 0.93). Black and gray arrows indicate significant and non-significant associations between the components, respectively. ^*^P < 0.05, ^**^P < 0.005, ^***^P < 0.001; CI: 95% bootstrap confidence interval for each of the standardized beta estimates.

We further examined whether the depressive symptoms of individuals also mediate the relationship between change in the perceived state of the pandemic and compulsory vs. voluntary motives to comply with prevention measures. All model specifics were set the same except that an outcome variable was replaced to the change in compulsory vs. voluntary motives from the change in the importance of social distancing. Based on previous studies about the importance of voluntary motives in facilitating highly sustained cooperation (Ryan and Deci, 2000; Cerasoli et al., 2014), we first set the compulsory relative individuals to voluntary motives as the outcome of interest (see Figure 4A; Supplementary Figure 4). Then, to expand our understanding of which motives were more heavily influenced by the belief change and depressive symptoms, we examined two separate mediation models, one with voluntary motives (see Figure 4B; Supplementary Figure 7) and the other with compulsory motives included as an outcome variable (see Supplementary Figures 5, 6).

To illustrate the interaction effect of the state of the pandemic and average preventive behavior in explaining the change of voluntary preventive motives, we analyzed the data from participants in the top 10% and bottom 10% in their average preventive behavior (Supplementary Figure 8). We calculated correlations between the beliefs of individuals about the state of the pandemic and the voluntary motives of the two groups.

### Trend Analyses

We used a two-sample *t*-test to compare whether objective states of the COVID-19 pandemic (i.e., number of new cases) changed between Time 1 and Time 2 data collection. Linear regression analyses were used to estimate the trends of viral transmission in South Korea, which confirmed that participants experienced a decreasing trend at Time 1 and an increasing trend at Time 2. The belief about the pandemic, behavioral intentions and motives, and depressive symptom severity was measured at each time point of data collection. Paired *t*-tests were used to test whether each measure changed between two time points. All statistical tests were two-tailed with an alpha level of 0.05 unless noted otherwise. SPSS software was used for the mediation analyses, and MATLAB R2019b was used for all the rest of the statistical tests.

## Results

### Individuals Update Their Beliefs About the COVID-19 Pandemic Following the Actual Change of the Pandemic State

We first examined the perception of the current pandemic state. Specifically, participants estimated how close they think it is to the end of the pandemic (0% = *initial outbreak*, 100% = *end of the pandemic*; see “Beliefs” in Materials and methods). Participants reported that the COVID-19 situation of Time 2 was at an earlier stage than that of Time 1 [Paired *t*-test, *t*_(1, 143)_ = 5.31, *P* = 1.33e-07; Figure 1B], showing that they updated their belief following the objective information. Such a change in belief was specific to the COVID-19 pandemic state in South Korea. Participants responded that the pandemic situation of other countries were proceeding toward later stage at Time 2 compared with Time 1 [*t*_(1143)_ = −7.76, *P* = 1.87e-14; Figure 1B]. Considering the comparable numbers of new cases at the two time points in South Korea, these results suggest that participants are sensitive to temporal trends of the pandemic and that they pay more attention to domestic situations than to foreign situations.

Such a belief about the state of the pandemic was significantly correlated with the concerns of individuals about being infected (see Materials and methods; Supplementary Figure 2). Particularly, both at Time 1 and Time 2, participants who believed South Korea to be further from the end of the pandemic (higher score indicates the belief of individuals that the pandemic is getting closer to the end) reported a higher risk of themselves being infected (Time 1: Pearson's correlation, *r* = −0.18, *P* = 8.79e-10; Time 2: *r* = −0.13, *P* = 1.76e-05; Figure 1C; Supplementary Figure 2). In other words, participants who perceived the situation severer believed that they were more likely to be infected. Based on this correlation between the perceived risk of getting infected and the COVID-19 pandemic state, one might expect that individuals would show greater compliance with prevention measures at Time 2 with the severer pandemic situation and higher risk of infection than Time 1. However, this was not the case, as shown in the following section.

### Voluntary Motives and Behavioral Intention to Follow Prevention Measures Diminished at a Second Wave

Using the measures of voluntary and compulsory motives (see “Motives” in Materials and Methods), we examined whether the motives of the individuals changed between Time 1 and Time 2. Mean ratings for voluntary motives did not change [Paired *t*-test, *t*_(1, 143)_ = 1.02, *P* = 0.31; Figure 2A], whereas mean ratings for compulsory motives increased from Time 1 to Time 2 [*t*_(1, 143)_ = −5.22, *P* = 2.18e-07; Figure 2B]. These results suggest the possibility that individuals become more dependent on compulsory motives as the COVID-19 situation lasts longer.

Consistent with the relative reduction of voluntary motives, participants reported a higher frequency of violating behaviors against social distancing at Time 2 than Time 1 (see Supplementary Material). Compared with Time 1, participants reported at Time 2 that they went out more often during the past week [Paired *t*-test, *t*_(1, 125)_ = −8.23, *P* = 5.06e-16; Figure 2C] and met more people during the past week [*t*_(1, 118)_ = −6.44, *P* = 1.73e-10; Figure 2D]. Similarly, the perceived importance of social distancing did not reflect the increased severity of the pandemic situation (or the belief update). The ratings for importance of social distancing (see “Behavioral intention” in Materials and Methods) remained the same on average [*t*_(1143)_ = 1.03, *P* = 0.31; Figure 2E].

The following section further investigated the mismatch between the change in beliefs and the change in behavioral intention. Here, we included the importance of social distancing as a measure of behavioral intention. This was because the direct preventive behaviors were confounded with the essential needs for leaving the house (and meeting other people) (e.g., going to work or visiting doctors) and could be susceptible to changes in local policies and social atmosphere.

Instead of including the direct measures in the mediation models, we performed correlation analysis to confirm that the importance of social distancing was associated with actual behaviors. As we expected, the importance of social distancing was significantly correlated with both the number of people participants met (Pearson's correlation r = −0.082, *P* = 0.0015) and the number of times they went out (*r* = −0.078, *P* = 0.0025; a negative correlation indicates consistency between measures) at Time 1. Yet, these correlations became non-significant at Time 2 (number of people: *r* = −0.030, *P* = 0.31; number of times: *r* = −0.044, *P* = 0.14), suggesting that the direct behavioral measures could be unstable across time. On the contrary, the importance of social distancing at Time 1 was significantly correlated with the average self-reported tendency to carry out preventive behaviors measured at Time 2 (the average tendency of individuals during the past 2 months from the time of the report; *r* = 0.27, *P* = 2.19e-20). This result indicates that our measure of behavioral intention at Time 1 is partly associated with the subsequently measured preventive behavior of individuals.

### Negative Belief Update Decreased Voluntary Motives and Behavioral Intention to Follow Prevention Measures via Depressive Symptoms

Our findings so far demonstrate that participants were responsive to the dynamically changing state of the COVID-19 pandemic. On the contrary, observed changes in their behavioral intention conflicted with how they updated their beliefs. In other words, participants who perceived the state of pandemic severer (further from the end) at Time 2 than Time 1 considered social distancing less important (*r* = 0.20, *P* = 3.76e-12; see Supplementary Figure 10). To address this mismatch, we examined the mediating role of the affective states of individuals. We conducted mediation analyses (Preacher and Hayes, 2004, 2008) with the perceived change of the COVID-19 pandemic state (Beliefs) as a predictor, the average preventive behavior of individuals during the past 2 months at Time 1 (Preventive behaviors) as a moderator, change in the importance rating for social distancing (Behavioral intention) as an outcome variable, change in depressive symptoms as a mediator, and sex and age as control variables (Figure 3; Supplementary Figure 3). Both direct (c_1_', Figure 3; Supplementary Figure 3) and indirect effects (${\text{a}}_{1}^{*}$b_1_, Figure 3; Supplementary Figure 3) were significant, indicating that the depressive symptom of individuals changes indeed mediated the relationship between their belief about the pandemic and their behavioral intention. Particularly, individuals who perceived the COVID-19 situation as severer at Time 2 compared with Time 1 reported greater depressive symptoms at Time 2 compared with Time 1, and individuals who experienced severer depressive symptoms at Time 2 than at Time 1 regarded social distancing as less important at Time 2 than at Time 1.

Notably, a similar relationship was found among the belief update, depressive symptoms, and motives to comply with prevention measures (Motives). The same mediation model with the relative contribution of compulsory vs. voluntary motives as a dependent variable revealed a significant indirect effect (Figure 4A). Particularly, participants who perceived the COVID-19 pandemic severer at Time 2 than at Time 1 became more dependent on compulsory than voluntary motives, and increased depressive symptoms mediated this relationship (Figure 4A; Supplementary Figure 4). Separate examination of the changes in voluntary (Figure 4B; Supplementary Figure 7) and compulsory (Supplementary Figures 5, 6) motives revealed that the increase in the relative contribution of compulsory vs. voluntary motives was mainly resulted from the relative decrease in voluntary motives. Consistent with previous findings on the relationship between affective states and voluntary motives (Isen and Reeve, 2005), individuals who became more depressed at Time 2 reported diminished voluntary motives for preventive behaviors. We also found a significant moderation effect of the preventive behavior of individuals on the association between their beliefs and voluntary motives (Supplementary Figure 8), which supports our hypothesis that individuals who had reasons for positive expectations (by complying with preventive behaviors) receive a larger impact from the unexpected negative outcomes (the pandemic getting worse).

## Discussion

Our data showed that individuals updated their beliefs following the continuously evolving COVID-19 situation. They correctly perceived the increasing phase of the second wave severer than the declining phase of the first wave. However, inconsistent with their beliefs, the perceived importance of social distancing did not increase, and motives to follow prevention measures shifted toward compulsory rather than voluntary motives. This finding suggests that the reduced compliance with government policies witnessed worldwide might not be due to inaccurate beliefs about the pandemic. Instead, such mismatch among belief, behavioral intention, and motives to comply with prevention measures seems to be mediated by changes in affective states in response to the worsening of the pandemic situation contrary to the expectations of individuals.

Under uncertain situations like the current COVID-19 pandemic, individuals constantly make predictions about future events and compare them with reality in order to update knowledge about the dynamically changing environment (Montague and Berns, 2002; O'doherty et al., 2003; Seymour et al., 2004; Behrens et al., 2007). Prediction errors (i.e., the difference between the expectation and observation) enable individuals to update their beliefs and adapt to the environment while being accompanied by affective experiences. For instance, positive and negative prediction errors involve positive and negative emotions, respectively (Villano et al., 2020). Our data support that individuals who experienced greater negative prediction error (i.e., greater change in belief) showed stronger affective responses (i.e., more depressed). This suggests that, in addition to the high level of stress from social isolation and fear of being infected (Arora et al., 2020; Brooks et al., 2020; Torales et al., 2020), the change of pandemic state in a negative direction and the corresponding change in individual belief can have negative impacts to mental health, even in the countries where relatively lower epidemic statistics are reported.

Another possible explanation could be that our findings reflect the psychological reactance against the uncontrollable COVID-19 situation of individuals. According to psychological reactance theory (Brehm and Brehm, 1981; Fogarty, 1997; Crawford et al., 2002; Rosenberg and Siegel, 2018), a situation that threatens or eliminates freedom induces negative effects and motivates people to restore their autonomy by engaging in forbidden or restricted behaviors. In line with this view, a recent study showed a “fatalism effect” that the information of experts experimentally manipulated to induce negative expectation error about the COVID-19 situation (e.g., higher risk of viral transmission than expected) decreased the intention to perform preventive behavior (Akesson et al., 2020; Jimenez et al., 2020). Consistently, the current study suggests that negative change in belief about the pandemic followed by negative affect results in a significant reduction of voluntary motives to comply with government policies. Given that voluntary than compulsory motivation is more efficient in facilitating and maintaining public cooperation (Ryan and Deci, 2000; Cerasoli et al., 2014), our findings highlight the importance of psychological factors that health agencies and government should consider when implementing preventive policies.

With the recent understanding of the COVID-19 pandemic acknowledging asymptomatic viral transmissions (around 45% of all cases) (Oran and Topol, 2020) and predicting a long-lasting endemic (Hunter, 2020), practicing personal prevention measures, including social distancing, seems to be consistently an important way to control the pandemic given the shortage of vaccines and the persistent threats of new variants of COVID-19 (Callaway, 2021; Moore and Offit, 2021). Such a restrictive range of control led government officials to come up with extra layers of enforced policies (e.g., South Korea launched a five-level social distancing scheme). This is worrisome because public cooperation enforced by external control is known to be more fragile than that by intrinsic motivation (Ryan and Deci, 2000; Cerasoli et al., 2014). An alarming result from the current study is that negative effect resulting from negative belief update reduced behavioral intention and voluntary motives to follow prevention measures. This implies that a prolonged pandemic situation combined with governmental norm enforcement may have triggered negative effects and reactance, followed by reduced voluntary motives, which would require more compulsory regulations. This chain of psychological responses should be carefully considered when government officials apply regulations (Arora et al., 2020).

There are a few limitations in the current study. First, it should be noted that the relationships between the variables in our mediation models are correlational. Although we hypothesized and tested the possibility where updates in the belief of individuals about the state of the pandemic precede other affective responses and intention changes, alternative causal relationships may exist as well. For example, depression might have yielded negative belief updates (the pandemic got worse), or stronger enforced compulsory motives might have made individuals even more depressed. Thus, causal directions should be interpreted with caution. Second, other possibilities may explain why individuals showed changes in their affective states, behavioral intentions, and motives. For example, individuals may feel powerless and experience learned helplessness when adhering to social distancing during the first wave yet got to experience a second wave (Khan et al., 2021). There is also a potential of psychological habituation (Ziferstein, 1967) at work, such that individuals became familiar with the situation and reported relatively less voluntary motives accordingly. These accounts, including the psychological reactance theory, are not mutually exclusive and cannot be ruled out in the current study design. Third, we cannot rule out the existence of ceiling effect in measuring the perceived importance of social distancing. The absence of changes in the perceived importance of social distancing between two time points could be partially due to the fact that individuals already perceived social distancing as highly important at Time 1 (mean = 86.39, STD = 16.02, range = [3–100]) and thus there might have been no room for a further increase at Time 2. Fourth, behavioral measures which we collected might be confounded with the changes in official policy for prevention measures. Although our *ex-post* analysis showed that the numbers of new cases were comparable between two time points, we cannot rule out potential impacts of policy changes that were only applied to particular regions with new outbreaks of cluster infections since May 6, 2020. Fifth, and lastly, there is a possibility that participants might have had insufficient evidence to increase preventive behaviors at Time 2 because they expected even severer pandemic situations. However, our data showed that, despite the comparable number of daily new cases, participants perceived Time 2 as a severer pandemic state than Time 1. This direction of change in subjective severity suggests that individuals are sensitive to the trend of change. Thus, it is unlikely that the diminished preventive intention and voluntary motives of individuals were due to insufficient evidence.

Nevertheless, our results suggest that psychological factors, including the affective and motivational states, should be considered in making policies to deal with the pandemic. For example, government officials might need to minimize uncertainty about the current pandemic status by planning efficient contact tracing and testing methods (Fiore et al., 2021) so that citizens could establish correct beliefs. At the same time, to promote voluntary cooperation from the people, we stress the risk of premature relaxation of prevention policies or overly optimistic information because the unexpectedly disappointing outcome may set off public resistance. Indeed, the COVID-19 pandemic status in South Korea worsened even further than the peak of the first wave (Bae, 2020). These implications could be extended to vaccination policies or a more general domain of public health policies. To sum up, our findings call attention to the importance of understanding psychological responses to the COVID-19 situation in devising policies to promote intrinsically motivated cooperation of the public for keeping their physical and mental health, and at last, to overcome the pandemic.

## Data Availability Statement

The datasets presented in this study can be found in online repositories. The names of the repository/repositories and accession number(s) can be found at: https://github.com/dongilchung/covid-depression-belief.

## Ethics Statement

The studies involving human participants were reviewed and approved by Institutional Review Boards of Ulsan National Institute of Science and Technology. The participants provided their written informed consent to participate in this study.

## Author Contributions

SS and DC: conceptualization, supervision, and funding acquisition. JP, SL, SS, and DC: methodology and writing—review and editing. JP and SL: formal analysis and investigation. JP and DC: visualization. JP, SS, and DC: writing—original draft. All authors contributed to the article and approved the submitted version.

## Conflict of Interest

The authors declare that the research was conducted in the absence of any commercial or financial relationships that could be construed as a potential conflict of interest.
