# Supplementary material for: Depression Symptoms Mediate Mismatch Between Perceived Severity of the COVID-19 Pandemic and Preventive Motives
Source: Front Psychol. 2021 Jul 22;12:650042. doi: 10.3389/fpsyg.2021.650042 (PMC8339883; doi:10.3389/fpsyg.2021.650042)
Supplement: Supplementary file 1 [file Data_Sheet_1.docx]

**Supplementary text**

**COVID-19 status in South Korea**

South Korea has experienced a drastic increase of new cases from February 18, 2020, of which origin was traced back to a patient number 31 who became a source of a large-scale viral transmission through religious gatherings (Normile, 2020). Aggressive testing and contact tracing, jointly with a social distancing policy (Park et al.) were immediately implemented, and by May 1, the number of daily new cases was successfully reduced to lower than ten (**Fig. 1a**). Based on the flattened pattern of the COVID-19 spread, the Korea Centers for Disease Control and Prevention (KCDC) announced the relaxation of social distancing rules starting from May 6. Most local businesses were reopened as before the COVID-19 and citizens were allowed to go back to their normal life, while being recommended to exercise ‘routine-life’ distancing (*e.g.*, when possible, particularly indoors, to keep distance from others, wear face masks, and wash hands frequently). Unfortunately, within a few days, another sustained upsurge of new cases was reported. This prompted the KCDC to officially announce that the country is experiencing a second wave.

The KCDC provides official information about the number of daily new confirmed cases and the contact traces so that every citizen can be updated with the most current situation of the pandemic at least once a day. To test whether participants update their belief about the COVID-19 in accordance with the information provided by the KCDC, we collected data at two time points that have comparable numbers of new cases with the opposite trends. *Ex post* analysis of the numbers of new cases confirmed that the severity of the COVID-19 in South Korea between the two time points was comparable (Two-sample t-test, *t*(13) = –1.12, *P* = 0.28; **Fig. 1a**). Note that the epidemiological trends between the two time points were in the opposite direction (Time1: slope = –0.91, Time2: slope = 0.73). The decreasing trend at Time1 might have created optimistic expectations for the end of the pandemic, while the second upsurge at Time2 might have yielded belief update and negative affective responses (*e.g.*, feeling depressed).

**Survey questions**

Objective state of the COVID-19

Objective severity of the COVID-19 pandemic was defined as the number of daily new confirmed cases (data obtained from <https://coronaboard.kr/>; **Fig. 1a**). The averages of daily new cases for the 7 days prior to the stated dates (gray solid line) depict the changing trend of the pandemic situation.

Likelihood of viral infection

We asked participants to estimate the likelihood of viral infection when the subject of infection was i) themselves, ii) family members, iii) friends and acquaintances, iv) neighbors, v) strangers from the same area of residence, or vi) strangers from different areas of residence. Infection likelihood was reported in an 11-point scale where each point from zero to 11 represents [no risk of infection (0%), 10, 20, …, and 100% probability of infection], respectively (**Fig. 1c, S1**).

Violating behaviors against social distancing

To measure the extent to which participants violated government suggested social distancing, we asked the following questions (**Fig. 2c, 2d**).

- How many times did you go out during the past one week?
- How many people did you interact with during the past one week?

Any entries that exceed the criteria of mean ± 3 standard deviations were excluded from data analyses; 18 and 25 data entries were excluded from the number of going-outs and the number of others interacted with, respectively.

Because South Korean government did not impose lockdown, answers to both questions could reflect either essential or non-essential (or both) reasons for going out. Thus, we asked participants to indicate specific reasons for going out among a list of essential (going to work, grocery shopping, visiting hospitals or drug stores, picking up take-out foods) and non-essential (social gathering, dining out or going to a pub, doing outdoor workouts, working out at a gym, visiting beauty salons) reasons. Participants’ responses were binary coded in further analyses based on whether they have been out for any non-essential reason (coded as 1) or not (coded as 0).

**Fig S1. Representativeness of the sample.** We intended to recruit a large number of sample nationally representative for sex, age, and areas of residence. Out of the original sample of 1500 individuals recruited at Time1, 1144 individuals (76.27%) remained opt-in for Time2. To examine whether both the original and the remained samples show appropriate levels of representativeness, we compared each set of sample against the population characteristics obtained from Korean Statistical Information Service (KOSIS; <https://kosis.kr>). All differences were under 2%, suggesting that our samples are nationally representative for gender, age, and area of residence in South Korea.

**Fig S2. Likelihood of viral infection.** Participants were asked to report their belief about probability of viral infection when the subject of the infection were individuals who are at six different levels of social distance: participants themselves (**Fig. 1c**), family members, close friends and acquaintances, neighbors, strangers from the same area of residence, and strangers from the different area of residence. Expected probability of viral infection was significantly higher at Time2 than Time1 for all cases (family Time1 (mean ± s.t.d.) = 26.74 ± 21.08, Time2 = 30.89 ± 21.59, t(1143) = –6.65, *P =* 4.45e-11; friends Time1 = 31.52 ± 20.75, Time2 = 35.65 ± 21.21; t(1143) = –6.60, *P =* 6.31e-11; neighbors Time1 = 34.06 ± 20.51, Time2 = 38.35 ± 21.37, t(1143) = –6.84, *P =* 1.25e-11; same area strangers Time1 = 37.95 ± 21.29, Time2 = 42.17 ± 21.80, t(1143) = –6.64, *P =* 4.88e-11; different area strangers Time1 = 42.92 ± 22.09, Time2 = 46.22 ± 22.30, t(1143) = –4.87, *P =* 1.25e-06). Note that the perceived likelihood of getting infected for others were significantly higher than that for themselves (Time1 = 25.78 ± 20.83, Time2 = 29.81 ± 21.41) at both time points. At both time points, individuals who reported that they are under high risk of viral infection reported greater concerns for others (Time1 Pearson’s correlations between self and family: r = 0.93, friends: r = 0.88, neighbors: r = 0.81, same area strangers: r = 0.60, different area strangers: r = 0.71; Time2 self and family: r = 0.93, friends: r = 0.86, neighbors: r = 0.80, same area strangers: r = 0.72, different area strangers: r = 0.63; all *P*s < 2.22e-16). Participants who showed greater increase in the perceived risk for self between the two time points also expected greater risk for others at Time2 than Time1, regardless of the subject (Pearson’s correlations between the changes in the perceived risk for self and family: r = 0.89, friends: r = 0.81, neighbors: r = 0.72, same area strangers: r = 0.62, different area strangers: r = 0.53; all *P*s < 2.22e-16). Shades represent estimated kernel density. The lower and upper hinges of box plots correspond to the first and third quantiles, and whiskers span the 95% confidence interval. Horizontal red and black lines indicate mean and median, respectively, and dots indicate outliers. Violin plots were generated using the ggplot2 R package (Wickham, 2016).

**Figure S3. Changes in individuals’ depressive symptom severity mediate the relationship between change in belief (and individuals’ tendency to follow preventive behavior) and change in perceived importance of social distancing.** To examine the mediation effect of change in depressive symptoms, **(a)** change in belief about the COVID-19 state between Time1 and Time2 and **(b)** individuals’ average preventive behaviors (e.g., wearing masks) at Time1 were set as predictors, and perceived importance of social distancing as an outcome variable. **(a)** Change in belief about the COVID-19 state was significantly associated with change in self-reported depressive symptoms (a_1_: *t* = –2.49, *P* = 0.013), such that individuals who believed the state of COVID-19 at Time2 was severer than at Time1 felt more depressed at Time2 than at Time1. Increase in depression symptom severity was significantly associated with decrease in the perceived importance of social distancing (b_1_: *t* = –3.64, *P* = 0.00028). The indirect effect derived from these associations (effect size = 0.0079, CI = [0.0010, 0.019]) in addition to the direct effect (c_1_’: *t* = 3.75, *P* = 0.00019) was significant. **(b)** The same mediation effect was observed when individuals’ tendency to follow preventive behaviors was included as a predictor. The tendency to follow preventive behaviors was positively correlated with increase in the depressive symptoms (a_1_: *t* = 2.46, *P* = 0.014). The increase in depression severity was correlated with decrease in the perceived importance of social distancing (b_1_: *t* = –3.64, *P* = 0. 00029). The indirect effect derived from these associations (effect size = –0.0080, CI = [–0.019, –0.0011]) in addition to the direct effect (c_1_’: *t* = *P* = 0.020) was significant. Black and gray arrows indicate significant and non-significant associations between the components, respectively. ^*^*P* < 0.05, ^***^*P* < 0.001; CI: 95% bootstrap confidence interval for each of the standardized beta estimates.

**Figure S4. Changes in individuals’ depressive symptom severity mediate the relationship between change in belief (and individuals’ tendency to follow preventive behavior) and compulsory vs. voluntary motives to follow prevention measures.** To examine the mediation effect of change in depressive symptoms, **(a)** change in belief about the COVID-19 state between Time1 and Time2 and **(b)** individuals’ average preventive behaviors (e.g., wearing masks) at Time1 were set as predictors, and compulsory versus voluntary motives were set as an outcome variable. **(a)** Individuals who believed the COVID-19 state was worse at Time2 than Time1 were more likely to show a greater increase in the self-reported depressive symptoms (a_1_: *t* = –2.47, *P* = 0.014). Increased depressive symptoms were associated with increased compulsory vs. voluntary motives [compulsory(Time2) – voluntary(Time2)] – [compulsory(Time1) – voluntary(Time1)] (b_1_: *t* = 3.71, *P* = 0.00022). The mediation effect of depressive symptom derived from these associations was significant (effect size = –0.0079, CI = [–0.017, –0.00090]), whereas the direct effect of the predictor on the outcome was not significant (c_1_’: *t* = –0.48, *P* = 0.63). **(b)** The tendency to follow preventive behaviors was positively correlated with increased depressive symptoms (a_1_: *t* = 2.46, *P* = 0.014). Increase in depressive symptoms were associated with increase in compulsory vs. voluntary motives [compulsory(Time2) – voluntary(Time2)] – [compulsory(Time1) – voluntary(Time1)] (b_1_: *t* = 3.70, *P* = 0.00023). The mediation effect of depressive symptoms derived from these associations were significant (effect size = 0.0081, CI = [0.0013, 0.017]), whereas the direct effect of predictor on the outcome was not significant (c_1_’: *t* = 0.66, *P* = 0.51). Black and gray arrows indicate significant and non-significant associations between the components, respectively. ^*^*P* < 0.05, ^***^*P* < 0.001; CI: 95% bootstrap confidence interval for each of the standardized beta estimates.

**Figure S5. Changes in individuals’ depression symptom severity do not mediate the relationship between change in belief about the COVID-19 state and reduction of compulsory motives to follow prevention measures.** To examine the moderated mediation effect of individuals’ depressive symptom, we set change in belief about the COVID-19 state between Time1 and Time2 as a predictor, individuals’ average preventive behavior (e.g., wearing masks) at Time1 as a moderator, and change in compulsory motives to follow prevention measures as an outcome variable. Individuals who believed the COVID-19 state was worse at Time2 than Time1 reported increased depression symptoms (a_1_: *t* = –2.40, *P* = 0.016). Individual tendency to follow preventive behavior before Time1 (a_2_: *t* = 2.38, *P* = 0.017; path not depicted) was also positively correlated with increased depression symptoms. However, there was no significant association between change in depression severity and change in compulsory motives (b_1_: *t* = 1.44, *P* = 0.15). The direct effect between belief change and change in compulsory motives (c_1_’: *t* = 2.56, *P* = 0.010) was significant even after adjusting for an effect of change in depressive symptoms. On the contrary, there was no significant association observed between average preventive behavior and change of compulsory motives (c_2_’: *t* = –1.70, *P* = 0.090; path not depicted). In addition, an interaction between change in belief about the COVID-19 state and average preventive behavior was not associated with change in depression symptoms (a_3_: *t* = –0.87, *P* = 0.93) and external motives (c_3_’: *t* = 0.37, *P* = 0.71). Black and gray arrows indicate significant and non-significant associations between the components, respectively. ^*^*P* < 0.05; CI: 95% bootstrap confidence interval for each of the standardized beta estimates.

**Figure S6. Change in individuals’ depressive symptom severity does not explain the relationship between change in belief about the COVID-19 state (and individuals’ tendency to follow preventive behavior) and compulsory motives to follow prevention measures.** To examine the mediation effect of change in depressive symptom, **(a)** belief about the COVID-19 state and **(b)** individuals’ average preventive behavior (e.g., wearing masks) were set as predictors, and compulsory motives were set as an outcome. **(a)** Individuals’ affective change between Time1 and 2 was significantly associated with state change (a_1_: *t* = –2.47, *P* = 0.014). However, depressive symptom severity was not associated with compulsory motive change (b_1_: *t* = 1.32, *P* = 0.19). Nevertheless, the direct effect was significant between compulsory motive alteration between Time1 and 2 and belief change about state of the COVID-19 (c_1_’: *t* = 2.58, *P* = 0.010). **(b)** Individuals’ affective change between Time1 and 2 was positively associated with average preventive behavior (a_1_: *t* = 2.46, *P* = 0.014), but not with compulsory motives (b_1_: *t* = 1.26, *P* = 0.21). There was no direct effect of average preventive behavior on compulsory motives either (c_1_’: *t* = –1.72, *P* = 0.086). Black and gray arrows indicate significant and non-significant associations between the components, respectively. ^*^*P* < 0.05, ^**^*P* < 0.005; CI: 95% bootstrap confidence interval for each of the standardized beta estimates.

**Figure S7. Individuals’ depressive symptom severity explains the relationship between initial belief (or preventive behavior) predictor and voluntary motives to follow prevention measures.** To examine the mediation effect of individuals’ depressive symptom on their beliefs and motives, **(a)** belief about state of the COVID-19 and **(b)** individuals’ average preventive behaviors (e.g., wearing masks) at Time1 were set as predictors, and perceived importance of social distancing was set as an outcome variable. **(a)** Individuals who believed the COVID-19 state was worse at Time2 than Time1 reported greater depressive symptoms at Time2 than at Time1 (a_1_: *t* = –2.47, *P* = 0.014). Increased depressive symptoms were associated with decreased voluntary motives (b_1_: *t* = –3.96, *P* = 0.00008). The indirect effect derived from these associations (effect size = 0.0084, CI = [0.0011, 0.019]) in addition to the direct effect (c_1_’: *t* = 5.39, *P* < 0.00001) was significant. **(b)** Individuals with a higher tendency to follow preventive behaviors at Time2 showed a greater increase in depressive symptoms (a_1_: *t* = 2.46, *P* = 0.014). The greater increase in depressive symptoms was correlated with lower voluntary motives at Time2 than at Time1 (b_1_: *t* = –4.03, *P* = 0.00006). The indirect effect derived from these associations (effect size = –0.0087, CI = [–0.018, –0.0016]) in addition to the direct effect (c_1_’: *t* = –4.06, *P* = 0.000053) was significant. Black and gray arrows indicate significant and non-significant associations between the components, respectively. ^*^*P* < 0.05, ^***^*P* < 0.001; CI: 95% bootstrap confidence interval for each of the standardized beta estimates.

**Figure S8. The association between belief about the COVID-19 pandemic state and voluntary motives is moderated by individuals’ tendency to follow preventive behavior.** As shown from the mediation model in **figure 4**, individuals who perceived the COVID-19 situation as severer at Time2 compared to Time1 (a_1_) and followed prevention measures more diligently at Time1 than others (a_2_) reported greater depressive symptoms at Time2 than at Time1. In addition, an interaction between the two predictors (*i.e.*, state of the COVID-19 pandemic and average preventive behavior) was associated with the motivational change. That is, individuals who did not follow preventive measures reported decreased voluntary motives as they perceived the pandemic was getting worse, while such an association was not found for the individuals who better practiced prevention behavior. To better illustrate the interaction effect, participants who were at the top and bottom 10% in their average preventive behaviors were selected as two subgroups. It turned out that the individuals with average prevention behaviors at the bottom 10% showed a significant positive correlation between changes in belief about the COVID-19 pandemic state and voluntary motives (r = 0.30, *P* = 0.0014), while such a pattern was not found among the individuals at the top 10% (r = 0.012, *P* = 0.90). This result implies that voluntary motives of individuals who strictly followed the prevention measures were not influenced by the change of the COVID-19 pandemic state. Note that these results were calculated just to ease the interpretation rather than making any additional inference.

**Fig S9. Changes in individuals’ concern about viral infection were not associated with changes in their behavioral intention or motives.** Abstract and statistical form of information regarding the COVID-19 might have influenced individuals’ behavioral intention and motives (Halpern et al.). Individuals who do not have firsthand or secondhand experience of infection may underestimate the importance of prevention measures (*e.g.*, ‘why should I sacrifice my freedom for the sake of strangers?’). However, according to our data, people who considered the pandemic severer reported a higher likelihood of themselves getting infected (Pearson’s correlation r = –0.067, *P* = 0.023). We also found that change in individuals’ concern about viral infection was not associated with their behavioral intention or motives, suggesting that risk underestimation does not underlie the reduced compliance. Specifically, changes in perceived likelihood of infection (perceived likelihood about themselves being infected) were not associated with either **(a)** changes in perceived importance of social distancing (Pearson’s correlation, r = 0.044, *P* = 0.14), **(b)** changes in voluntary motives (r = 0.012, *P* = 0.68), or **(c)** changes in compulsory motives to follow prevention measures (r = 0.042, *P* = 0.15). Each dot indicates an individual participant.

**Fig S10. Correlations between variables-of-interest.** Correlations between all the major variables were computed and depicted as a confusion matrix. Each of the variables is the difference between measures that were collected from two time points (Time2 – Time1). Numbers denoted on the cells indicate Pearson’s correlation coefficients; any associations that are significant (*P* < 0.0011 = 0.05/45; applying Bonferroni correction for multiple comparisons) are in red.

**Figure S11. Alternative models: Time1 pandemic state as a predictor.** We examined the explanatory power of alternative models where individuals’ belief about the state of the pandemic self-reported at Time1 is set as a predictor instead of the difference measure between two time points. In all the four models-of-interest (testing impacts on importance of social distancing, compulsory relative to voluntary motives, and each of the motive separately), mediator, moderator, and outcome variable were set the same as the original models. This alternative predictor (belief about the state of the pandemic at Time1) showed significant association with **(a)**changes in importance of social distancing, **(c)** changes in compulsory motive, and **(d)** changes in voluntary motive. However, in all four models, no significant mediation or moderated mediation effects were observed among the variables.

**Fig S12. Alternative models: Time2 pandemic state as a predictor.** We examined the explanatory power of alternative models where individuals’ belief about the state of the pandemic self-reported at Time2 is set as a predictor instead of the difference measure between two time points. In all of the four models-of-interest (testing impacts on importance of social distancing, compulsory relative to voluntary motives, and each of the motive separately), mediator, moderator, and outcome variable were set the same as the original models. This alternative predictor (belief about the state of the pandemic at Time2) showed significant association with **(a)** changes in importance of social distancing and **(d)** changes in voluntary motive. However, in all of the four models, no significant mediation or moderated mediation effects were observed among the variables.

**Table S1. Demographic information of participants who completed both time points and individuals’ self-reported measures to variables-of-interest at each time point.**

|  | Time1 | Time2 | t-value | *P* |
| --- | --- | --- | --- | --- |
| Sex [male/female] | 583 / 561 | |  |  |
| Age | 45.04 (13.33) | |  |  |
| Tendency to carry out preventive behaviors^a^ | 6.05 (0.84) | 5.92 (0.90) | 3.42 | 6.45e-04 |
| Compulsory motives^b^ | 3.63 (1.78) | 3.91 (1.75) | -5.22 | 2.18e-07 |
| Voluntary motives^b^ | 5.90 (1.02) | 5.90 (1.01) | 1.02 | 0.31 |
| Importance of social distancing (%)^c^ | 86.39 (16.02) | 85.87 (16.34) | 1.03 | 0.31 |
| State of the COVID-19 pandemic (South Korea) (%)^d^ | 64.16 (18.58) | 60.62 (18.46) | 5.31 | 1.33e-07 |
| State of the COVID-19 pandemic (foreign countries) (%)^d^ | 43.08 (21.89) | 49.09 (20.83) | -7.76 | 1.86e-14 |
| Likelihood of infection (%)^e^ | 25.78 (20.83) | 29.81 (21.41) | -6.42 | 2.02e-10 |
| # of times individuals went out during the past one week | 3.76 (3.30) | 4.28 (2.64) | -8.23 | 5.06e-16 |
| # of people individuals met during the past one week | 13.65 (31.85) | 14.46 (18.26) | -6.44 | 1.73e-10 |
| Self-rating depression scale^f^ | 42.59 (8.88) | 42.68 (8.73) | -0.50 | 0.62 |

Standard deviation of each variable is given in parentheses; ^a^Measured in a 7-point Likert scale (1 = never, 7 = very frequently); ^b^Measured in a 7-point Likert scale (1 = definitely not, 7 = definitely); ^c^Measured in percentages from 0% (not important at all) to 100% (absolutely important); ^d^Measured in percentages from 0% (beginning) to 100% (complete end); ^e^Measured in a 11-point scale (0 = 0% risk of infection, 10 = 100% risk of infection); ^f^Zung Self-Rating Depression Scale (Zung, 1965).

**Table S2. Drop-out analysis: Comparison of demographic information and variables-of-interest measured at Time1 between participants who completed both time points and those who dropped out after Time1.**

|  | Time1 only  (N = 356) | Both Time1, 2 (N = 1144) | Statistics^g^ | *P* |
| --- | --- | --- | --- | --- |
| Sex [male/female] | 180 / 176 | 583 / 561 | χ^2^=0.017 | 0.90 |
| Age | 43.45 (13.09) | 45.04 (13.33) | -1.98 | 0.048 |
| Tendency to carry out preventive behaviors^a^ | 6.04 (0.93) | 6.05 (0.84) | -0.19 | 0.85 |
| Compulsory motives^b^ | 3.53 (1.80) | 3.63 (1.78) | -0.96 | 0.34 |
| Voluntary motives^b^ | 5.99 (0.98) | 5.90 (1.02) | 1.52 | 0.13 |
| Importance of social distancing (%)^c^ | 85.19 (17.90) | 86.39 (16.02) | -1.20 | 0.23 |
| State of the COVID-19 pandemic (South Korea) (%)^d^ | 63.56 (18.56) | 64.16 (18.58) | -0.54 | 0.59 |
| State of the COVID-19 pandemic (foreign countries) (%)^d^ | 43.43 (22.91) | 43.08 (21.89) | 0.25 | 0.80 |
| Likelihood of infection (%)^e^ | 24.35 (20.85) | 25.78 (20.83) | -1.13 | 0.26 |
| # of times individuals went out during the past one week | 3.97 (3.77) | 3.76 (3.30) | 1.02 | 0.31 |
| # of people individuals met during the past one week | 17.20 (49.67) | 13.65 (31.85) | 0.99 | 0.32 |
| Self-rating depression scale^f^ | 41.89 (8.33) | 42.59 (8.88) | -1.32 | 0.19 |

The second column (Time1 data from participants who completed both time points) is a duplicate of the first column of **Table S1**. Standard deviation of each variable is given in parentheses; ^a^Measured in a 7-point Likert scale (1 = never, 7 = very frequently); ^b^Measured in a 7-point Likert scale (1 = definitely not, 7 = definitely); ^c^Measured in percentages from 0% (not important at all) to 100% (absolutely important); ^d^Measured in percentages from 0% (beginning) to 100% (complete end); ^e^Measured in a 11-point scale (0 = 0% risk of infection, 10 = 100% risk of infection); ^f^Zung Self-Rating Depression Scale (Zung, 1965); ^g^Chi-square value is reported for sex, and t-values are reported for all other variables.

**Supplementary References**

Halpern, S.D., Truog, R.D., and Miller, F.G. Cognitive Bias and Public Health Policy During the COVID-19 Pandemic. *JAMA* 324**,** 337-338.

Normile, D. (2020). *Coronavirus cases have dropped sharply in South Korea. What’s the secret to its success* [Online]. Available: <https://www.sciencemag.org/news/2020/03/coronavirus-cases-have-dropped-sharply-south-korea-whats-secret-its-success> [Accessed 03 17].

Park, Y.J., Choe, Y.J., Park, O., Park, S.Y., Kim, Y.-M., Kim, J., Kweon, S., Woo, Y., Gwack, J., and Kim, S.S. Contact Tracing during Coronavirus Disease Outbreak, South Korea, 2020. *Emerging infectious diseases* 26.10.

Wickham, H. (2016). *ggplot2: elegant graphics for data analysis.* springer.

Zung, W.W. (1965). A self-rating depression scale. *Archives of general psychiatry* 12**,** 63-70.
